# Do Changes in the Local Food Environment Within New Residential Developments Influence the Diets of Residents? Longitudinal Results from RESIDE

**DOI:** 10.3390/ijerph17186778

**Published:** 2020-09-17

**Authors:** Alexia Bivoltsis, Gina Trapp, Matthew Knuiman, Paula Hooper, Gina L. Ambrosini

**Affiliations:** 1 School of Population and Global Health, The University of Western Australia, 35 Stirling Highway, Crawley, WA 6009, Australia; gina.trapp@telethonkids.org.au (G.T.); matthew.knuiman@uwa.edu.au (M.K.); gina.ambrosini@health.wa.gov.au (G.L.A.); 2 Telethon Kids Institute, PO Box 855, West Perth, WA 6872, Australia; 3 Australian Urban Design Centre, School of Design, The University of Western Australia, 1002 Hay Street, Perth, WA 6000, Australia; paula.hooper@uwa.edu.au

**Keywords:** longitudinal, food environment, diet

## Abstract

*Background*: There is limited longitudinal evidence supporting a link between food outlet locations and dietary outcomes to inform policy and urban planning. This study examined how longitudinal changes in the local food environment within new residential developments influenced changes in adult dietary intake. *Methods*: Adult participant data (*n* = 3223 person-observations) were sourced from the RESIDential Environments (RESIDE) project across three time points between 2004 to 2012 in Perth, Western Australia. Fixed effects regression estimated the relationship between change in spatial exposure to the local food environment, individual behaviours and perceptions of the local food environment with dietary outcome variables (healthy diet score, unhealthy diet score, diet quality score and fruit/vegetable intake). *Results*: An increase over time in the percentage of healthy food outlets around the home was significantly (*p* ≤ 0.05) associated with an increase in healthy diet scores and an increase in the distance from home to the nearest café restaurant was significantly (*p* ≤ 0.05) associated with an increase in diet quality scores. *Conclusions*: Modifying the local food environment by increasing the relative proportion of healthy food outlets around the home may support healthier dietary intake.

## 1. Introduction

Poor diet is a leading preventable risk factor contributing to the rise in obesity and nutrition-related chronic diseases (NRCD) such as cardiovascular disease, type 2 diabetes, and some cancers, both globally and in Australia [1,2,3,4]. Improving population dietary intakes can significantly reduce the economic and health burden associated with NRCDs [5,6]. An individual’s diet is shaped by their surrounding environment [7,8]. The local food environment (i.e., the spatial location, type and mix of food outlets around the home) can influence people’s diets by altering individual behaviours such as food outlet choice and purchasing patterns [9]. For example, living in an area with a greater density and proximity to fast food, takeaway outlets, café restaurants and convenience stores selling mostly processed, energy-dense foods, may promote unhealthy dietary intakes [10,11,12], whilst healthy food outlets selling fresh produce, fruit and vegetables (i.e., supermarkets and greengrocers) may support healthy diets [13]. Therefore, modifying the location, type and mix of food outlets may contribute to improving people’s diets at a population-level. Indeed, evidence suggests population-level interventions that take a regulatory approach beyond individual behaviour change are likely to be the most successful at reducing the current rise in obesity and NRCDs [14,15].

Despite this, in Australia, there is a lack of implementation of internationally recommended policies for addressing unhealthy diets and obesity via environmental-level interventions [16]. Planning policies that incorporate health as a key consideration are being recognised as an important step towards addressing the current rise in obesity and NRCD [17]. Yet, there has been limited progress towards implementing these recommendations [15]. Changes to planning laws that place restrictions on the locations of certain unhealthy food outlets (e.g., fast food outlets) whilst also setting targets regarding the inclusion of healthy food outlets (e.g., supermarkets and greengrocers) relies upon robust evidence to inform specific interventions. However, most of the evidence to-date is based on cross-sectional studies with inconclusive and often contrasting findings [18,19]. The largely cross-sectional literature cannot account for the dynamic relationships between the environment and diet, given that both the local food environment and individual-level factors evolve over time. Whilst research investigating changes in the local food environment following residential relocation show some influence on dietary outcomes [20], these findings do not provide insight into how diet will improve following modifications to the local food environment around stationary residents—which is the assumption of urban planning policies. Such longitudinal studies are limited and restricted to the US [21,22,23,24].

This study uses longitudinal data from the RESIDential Environments (RESIDE) project, a natural experiment conducted in Perth, Western Australia from 2003–2012 aimed at quantifying the health impact of urban design policy within new residential developments. RESIDE provided the unique opportunity to follow the evolution of local food environments within new residential developments and how these changes contribute to changes in dietary intakes. The aims were to (1) examine how longitudinal changes in spatial exposure to the local food environment, individual behaviours and perceptions of the local food environment within new residential developments influences changes in adult dietary intake, and (2) investigate scale effects via sensitivity analyses. The findings will provide stronger, localised evidence for targeted urban planning policy.

## 2. Materials and Methods

### 2.1. Study Sample and Data Collection

The RESIDential Environments (RESIDE) project is a longitudinal natural experiment that collected data between 2003 and 2012 from people who relocated from their home within an established neighbourhood into one of 73 new residential developments across Perth, Western Australia (WA). New developments were located within previously undeveloped greenfield or large brownfield sites (i.e., urban infill areas). A full description of new development characteristics is provided elsewhere [25,26,27].

Details of participant recruitment procedures have been reported elsewhere [28]. Briefly, households identified as building homes within new developments were invited to participate (response rate 33.4%) [28]. One person was randomly selected from each household and completed a self-reported questionnaire on physical activity, health, lifestyle behaviours, perceptions, usual food intake and socio-demographic variables at four time points; T1 prior to relocating (baseline: 2003–2005), T2 (1–2-years post move: 2004–2006), T3 (2–3 years post move: 2006–2008), and T4 (6–9 years post move: 2011–2012). In total, 1811 adults were recruited into the study at baseline.

The current study draws data from *n* = 1467; 1230 and 565 participants at T2, T3 and T4, respectively and represents people living in the same house within a new residential development. Person-observations with missing data at each time point were excluded including *n* = 14 at T2, *n* = 18 at T3, and *n* = 7 at T4. The final analytical sample was *n* = 1453; 1212 and 558 at T2, T3 and T4, respectively (*n* = 3223 person-observations). The date of questionnaire completion ranged from July 2004 to February 2007 for T2, October 2006 to December 2008 for T3 and February 2011 to March 2012 for T4. Ethics approval was provided by the University of Western Australia’s Human Research Ethics Committee (#RA/4/1/479).

### 2.2. Dietary Outcomes

The dietary outcome variables examined were a healthy diet score, an unhealthy diet score, a diet quality score and the frequency of fruit and vegetable intake. Details of the methods undertaken to derive the dietary outcome variables have been described previously [29]. In brief, at each time point participants were asked six questions: (1) How many servings of vegetables do you usually eat each day (including fresh, frozen and tinned)? (2) How many servings of fruit do you usually eat each day (including fresh, dried, frozen and tinned fruit)? (3) How often do you eat red meat (beef, lamb, and kidney but not pork or ham) including all minimally processed forms of red meat such as chops, steaks, roasts, rissoles, mince, stir-fries, and casseroles? (4) How often do you eat chips, French fries, wedges, fried potatoes or crisps? (5) How often do you eat meat products such as sausages, frankfurters, polony (bologna sausage), meat pies, bacon or ham? and (6) What type of milk do you usually consume? Fruit and vegetable intake were rated on a scale from 0–5 (0 = do not eat to 5 = 6 servings or more). The frequency of intake for items 3, 4, and 5 were rated from 0–6 (0 = never to 6 = most days, i.e., 6–7 days per week). Item 6, milk type, was coded 0 = whole (full cream), 1 = other (soy, lactose free, low or reduced fat, don’t drink milk), and 2 = skim. The raw frequencies of those foods recommended by the Australian Dietary Guidelines [30] to increase in the diet (items 1, 2 and 6) were summed to create a ‘healthy’ component score (range = 0–12) with higher numbers reflecting a healthier diet. The raw frequencies of those foods recommended to limit in the diet (items 3, 4 and 5) were summed to create an ‘unhealthy’ component score (range = 0–18) with higher numbers reflecting an unhealthier diet. The raw frequency categories for fruit and vegetable intake were also summed to create a single measure of fruit and vegetable intake (range = 0–10).

Given there were only six dietary questionnaire items consistently assessed across T2, T3 and T4, it was necessary to obtain a longitudinal measure of diet quality based on the available variables. Using the above six dietary questionnaire items, and a previously described approach [29], an *a priori* overall diet quality score (the simple RESIDE dietary guideline index or S-RDGI1) was calculated to assess overall diet quality in this study across T2, T3 and T4. Briefly, at T4 a diet quality index (RDGI) was derived using the most comprehensive dietary data available (24 items). A multiple linear regression model was then fitted using the RDGI scores (dependent variable) and the scores of the six dietary questionnaire items (independent variables), from which the estimated regression equation was used to predict the dependent variables (S-RDGI1) at T2, T3 and T4 when only the independent variables (scores of the six dietary questionnaire items) were known. Diet quality scores ranged from 0–100 with higher scores reflecting a better diet quality.

### 2.3. Spatial Exposure to the Local Food Environment

The locations of food outlets were sourced from a commercial database (SENSIS Pty. Ltd., Melbourne, Australia) at temporally matched time points of 2006 (T2), 2007 (T3), and 2011 (T4). Validation studies indicated moderate to good agreement between commercial listings and in situ locations of food outlets [31]. The methods undertaken to classify food outlets and measure individual, spatial exposure to the local food environment have been reported elsewhere [20,25]. Briefly, a range of spatial exposure measures were generated for the geocoded residential addresses of participants at T2, T3, and T4 including the road network distance to nearest (km) and a count within a 1.6 km road network buffer around the home of takeaway/fast food, café restaurants, convenience stores and supermarket/greengrocers. Lastly, a composite measure known as the modified retail food environment index (MRFEI) was derived using the count of all healthy food outlets divided by the count of all food outlets present, within a 1.6 km road network buffer around the home and multiplied by 100. A higher MRFEI (%) represents a greater relative percentage of ‘healthy’ food outlets and therefore a ‘healthier’ food environment. A 1.6 km road network buffer was chosen to reflect the conceptualisation of a ‘neighbourhood’ within the RESIDE study and represents a 15-min walk (30-min round trip) [32] known to capture 95% of usual walking destinations [33]. Furthermore, road network buffers may capture outlets accessible by walking more effectively than Euclidean buffers [34]. Sensitivity analyses were run using 0.8 km and 5 km road network buffers to investigate possible scale effects, given that over 90% of participants had access to a motor vehicle, and food purchase is likely to occur at distances greater than 1.6 km [35] and previous RESIDE findings demonstrated that having a supermarket within 0.8 km of home by road was associated with a healthy eating score at T4 [36]. All spatial analyses were undertaken using ArcGIS Desktop Version 10.5.1. Environmental Systems Research Institute (ESRI, Redlands, CA, USA).

### 2.4. Individual Behaviours

Participants were asked two questions on a 7-point scale (0 = never to 6 = 6–7 times per week) “How often do you eat meals that are bought from a canteen or takeaway food shop” and “How often do you eat meals that are bought from a restaurant or café”. Reliability of these items was high with test-retest reliability determined via intraclass correlations of 0.82 and 0.83, respectively [37,38].

### 2.5. Perceptions of the Local Food Environment

Information describing the way participants perceived their surrounding local food environment was obtained from responses to survey items based on the Neighbourhood Environment and Walking Scale questionnaire [39]. Participants were asked: “About how long would it take to get from your home to the nearest café or restaurant; greengrocer, and supermarket if you walked to them?” This information was then used to create two binary variables (yes, no) for perception of a café/restaurant or supermarket/greengrocer within a 15-min walk of home.

### 2.6. Adjustment Variables

All analyses were adjusted for time-varying participant characteristics including age, education level, marital status, hours of work per week, household income, children <18 years at home, access to a motor vehicle, physical activity, and body mass index (BMI) (see Table 1). A time-varying measure of area-level socio-economic status (SES) was assigned to each participant using the Australian Bureau of Statistics (ABS) 2006 Census Collection District (CCD) and 2011 Statistical Area Level 1 (SA1) Index of Relative Socioeconomic Advantage and Disadvantage (IRSAD). The IRSAD is derived from 21 Census variables related to income, education, employment, occupation and housing and represents a continuum of advantage (high values) to disadvantage (low values) [40]. The ABS applied deciles are an ordered scale from 1 (lowest 10%) to 10 (highest 10%). An individual’s area-level SES was the IRSAD decile value of the CCD (T2) or SA1 (T4) that fell under their residential address with an average of the 2006 and 2011 IRSAD values determined for each address at T3.

### 2.7. Statistical Analysis

Summary statistics were calculated for the analytical sample at T2, T3 and T4. This study aimed to quantify the within-person associations between changing local food environments, individual behaviours and perceptions with changing dietary outcomes. Therefore, generalised linear models (GLM) were used to fit “fixed subject effects” models to estimate the separate effect of change in each predictor variable (i.e., spatial exposure to the local food environment, individual behaviours and perceptions) on change in dietary outcomes (i.e., healthy diet, unhealthy diet, fruit/vegetable intake and diet quality). This model obtains a pure within-person estimate because it includes a fixed effect for each participant (i.e., a coefficient estimate), such that each participant acts as his/her own control for all measured and unmeasured time-constant variables. Models were adjusted for all time-varying participant characteristics and time. All statistical analyses were conducted using IBM SPSS Statistics for Windows, Version 23.0 (IBM Corp., Armonk, NY, USA).

## 3. Results

### 3.1. Summary Statistics

Table 1 presents summary statistics of participant characteristics for the analytical sample at T2, T3 and T4. Overall, the recruited study sample at T2 to T4 was slightly older, more affluent and with a higher percentage being female than the population of greater Perth (known as the Perth metropolitan area as defined by the Australian Bureau Statistics, 2012) [41]. Across the study period from T2 to T4 the number of people with a household income greater than $90,000 increased, as did the number of people with an education level of Bachelor or higher. Physical activity (hrs/week), BMI (kg/m^2^) and area-level SES (deciles) also increased slightly across the study period.

Table 2 outlines summary statistics of dietary outcomes and predictor variables for the analytical sample at T2, T3 and T4. Overall, there was a general improvement in all dietary outcomes from T2 to T4, however this was minimal. The frequency of eating meals bought from a canteen or takeaway food shop, or from a restaurant or café decreased slightly over time, whilst the percentage of people who perceived there to be a café or restaurant, or supermarket/greengrocer within 15-min walk of home increased. The count of all food outlet types increased gradually over time, as did the MRFEI, indicating a slight increase in the percentage of healthy food outlets from T2 to T4 and a general improvement in the healthiness of the local food environment. The distances from home to the nearest food outlet, for all outlet types, decreased slightly over time. These changes over time in the local food environment of new residential developments are consistent with previously published findings from the RESIDE study [26].

### 3.2. Associations between Changes in Predictor Variables and Changes in Dietary Outcomes

The single factor associations between study predictor variables and dietary outcomes are shown in Table 3. An increase in the percentage of healthy food outlets around the home (MRFEI) was significantly associated with an increase in healthy diet scores (β = 0.003; 95% CI 0.00, 0.01) and an increase in the road network distance from home to the nearest café or restaurant was significantly associated with an increase in diet quality scores (β = 0.24; 95% CI 0.01, 0.48). A one unit increase in the frequency of eating meals bought from a canteen or takeaway food shop was significantly associated with an increase in unhealthy diet scores (β = 0.37; 95% CI 0.29, 0.45), and decrease in healthy diet scores (β = −0.09; 95% CI −0.14, −0.05), fruit/vegetable intake (β = −0.10; 95% CI −0.14, −0.05), and diet quality scores (β = −0.67; 95% CI −0.88, −0.46). 

A one unit increase in the frequency of eating meals bought from a restaurant or café was significantly associated with an increase in unhealthy diet scores (β = 0.29; 95% CI 0.20, 0.39) and a decrease in fruit/vegetable intake (β = −0.07; 95% CI −0.12, −0.02) and diet quality scores (β = −0.52; 95% CI −0.76, −0.28). Participants who perceived there to be a café or restaurant within a 15-min walk of home had significantly greater fruit/vegetable intake (β = 0.11; 95% CI 0.001, 0.23).

### 3.3. Sensitivity Analyses

There were no significant associations with dietary outcomes for analyses using road network buffers of 0.8 km and 5 km (results not shown), with the exception of a decline in diet quality with an increase in café restaurants within 0.8 km buffers (β −0.50; 95% CI −0.92, −0.07; *p* = 0.022).

## 4. Discussion

### 4.1. Key Findings

This study used longitudinal data from three time points to examine how changes in individual behaviours and perceptions, along with spatial exposure to food outlets around the home, contributed to changes in dietary outcomes. Given existing evidence is almost exclusively cross-sectional [42], the findings from this study provide stronger, longitudinal evidence that living in a healthier local food environment supports better dietary outcomes.

These results are consistent with those of a previous study [20], which found that moving to a neighbourhood with a greater percentage of healthy food outlets around the home was associated with an increase in healthy food and fruit/vegetable intake—adding to evidence that relative measures of the food environment may be a more accurate predictor of dietary outcomes than absolute measures [43,44]. Similar findings have been reported in cross-sectional studies from Australia in which healthier dietary outcomes (i.e., fruit/vegetable intake and higher diet quality scores) were associated with relatively healthier food environment [13,45]. Indeed, other work has confirmed that relative and absolute measures assess different aspects of the food environment [46].

Present findings indicate increasing proximity to a café or restaurant may contribute to a decline in diet quality. This suggests that these food outlet types are likely to provide less healthy food choices. Whilst the food items sold within café restaurants may vary from country to country and among different outlets [47], within Australia, portion sizes of meals served at restaurants are often greater than recommended guidelines contributing to excess energy intake [48]. The same can be said for other countries. For example, in Canada the levels of saturated fat, sodium and sugar in restaurant meals is often up to 50% of recommended daily intakes [49]. Within the Australian literature, associations between less healthy food outlets (i.e., fast food outlets, restaurants and takeaways) and dietary outcomes in adults are mixed with the majority of studies reporting null findings [11,50,51,52,53]. One study in India found participants with a greater density of full-service restaurants were more likely to be obese and have poorer dietary outcomes [54]. In contrast, other studies in Brazil [55], and Japan [56] found no association between proximity to restaurants and overweight/obesity. However, this comes from a largely cross-sectional evidence base. Previous longitudinal research investigating the relationship between unhealthy food outlets (i.e., fast food) and diet quality suggest that these relationships may be understated in the literature due to residual confounding [22]. Longitudinal findings from the US have shown higher numbers of fast food restaurants around the home were associated with lower diet quality and increased fast food consumption [22,24].

Based on the sensitivity analyses, the findings in this study appear to be specific to the 1.6 km road network buffers around the home. One exception was a decline in diet quality following an increase in the number of café restaurants within 0.8 km buffers. The influence of these food outlets may be more apparent when situated closer to the home. Previous work has identified several scale effects on derived measures of spatial exposure to the local food environment [57,58], highlighting the importance of defining clear conceptual relationships between exposure and outcome measures. Within the Australian literature, studies have explored spatial scales ranging from 0.4 to 5 km [42]. However, for unhealthy food outlets, few Australian studies have investigated scale effects on adults [42]. In children, greater densities of fast food outlets and convenience stores within 0.8 km from home were associated with reduced fruit and vegetable intake [59]. Numerous contextual factors are likely to be influencing the spatial scale of relationships between food outlets and diet. For example, individual factors such as mobility (i.e., car ownership, public transport) and food shopping behaviours, along with environmental factors including the density and distribution of food outlets. Furthermore, whilst the proximity of food outlets to the home may play a role in influencing food intake, food is consumed in a wide range of setting (e.g., workplaces) and the surrounding environments of these settings may also influence dietary outcomes.

In contrast, this study found that people who perceived there to be a café or restaurant within a 15-min walk of their home had a higher fruit/vegetable intake. Whilst this may seem counterintuitive, perception-based measures are subjective and affected by individual factors. Research has shown weak correlations between perception-based measures and measures of spatial exposure of the local food environment [60]. In the present study, it may be that perceived closeness to a café or restaurant is a proxy for other individual behaviours or constructs for healthy food availability.

Increasing the frequency of eating meals from a canteen, takeaway food shop, restaurant or café was associated with a general decline in dietary outcome variables, confirming that eating behaviours have a direct effect on dietary intake. Indeed, frequently eating meals outside the home has been associated with poorer dietary and weight outcomes in previous research [61,62,63]. Therefore, it seems plausible that changes in the location, number and mix of food outlets may be driving changes in the way people utilise such food outlets, influencing diet. Whilst it is possible that behavioural variables such as the frequency of eating meals from a canteen, takeaway food shop, restaurant or café may act as mediators on the pathway between spatial exposure to food outlets and dietary intake, previous work found behavioural variables only slightly attenuated these relationships [20]. In the present study, the statistically significant relationships between spatial exposure to food outlets and diet were not considered sufficiently robust to support mediation analyses.

### 4.2. Implications for Policy and Planning

The findings from this study contribute important, robust local evidence that changing the local community food environment can contribute to changes in the diets of residents. This has implications for future urban planning and policies aimed at improving population dietary outcomes. Evidence suggests that modifying the local food environment by increasing the relative proportion of healthy food outlets around the home may support healthier dietary intake. It has been previously acknowledged [22] that interventions focusing on absolute changes to food outlets show little effect on diet [64,65,66,67,68,69], prompting an alternative approach. Moreover, findings suggest that limiting the density of café restaurants in close proximity to the home may also improve dietary outcomes. State and local governments can support the development of healthy local food environments via regulatory reforms to the planning legislation at the state level and changes to local government planning schemes. Targeting behaviours associated with eating outside the home may contribute to improving the diets of residents. Modifications to marketing, advertising, food labelling and price may enable healthier food choices outside the home [70]. Similarly, interventions that modify the healthfulness of food items provided at cafes and restaurants may also serve to improve population dietary outcomes. Such interventions may include promoting healthier food choices, providing relatively more healthy food options, labelling of unhealthy food options, as well as economic incentives to increase the attractiveness of healthier food choices.

### 4.3. Strengths and Limitations

This is the first Australian study to examine changes in the local food environment and diet over time, in the form of a natural experiment. The findings are supported by the use of multiple dietary outcomes, a range of diverse spatial exposure measures, individual residential address details and sensitivity analyses to test for scale effects. Despite these strengths, there are also study limitations, most of which were inherently related to the study design and have been previously discussed [20]. In brief these included: self-reported dietary intake; commercially sourced food outlet locations; place-based spatial exposure to the local food environment limited to around the home; lack of inclusion of food purchasing behaviour; and an absence of consumer food environment data. Lastly, whilst this study utilised fixed effects regression to account for all measured and unmeasured time-invariant confounding, there remains the potential for bias associated with changing unmeasured variables [22]. On a final note, given this research was conducted between 2004 to 2012, the changing landscape of food access and availability (i.e., developments in online food delivery methods) may serve to alter the observed relationships between exposure and outcome variables. Yet, the findings still offer valuable insight into how diet will improve following changes to the local food environment around stationary residents.

## 5. Conclusions

The results of this study provide strong, longitudinal evidence that changes to the local food environment around the home to include a greater relative proportion of healthy food outlets can lead to improved population dietary intakes. These findings are also likely to be sensitive to scale, as results were only significant within a 1.6 km road network buffer around the home. Policy approaches that focus on increasing the number of healthy food outlets, whilst limiting unhealthy food outlets may therefore be effective. Furthermore, findings suggest that increasing the distance from home to the nearest café or restaurant may improve diet quality. Targeting both the healthiness of food sold in cafes and restaurants along with factors influencing the frequency of eating meals outside the home may be an important policy approach. However, more research is needed to better understand the mechanisms via which spatial exposure to food outlets influences dietary intake, by exploring the potential of mediating and moderating effects such as education level, income, having children in the home, purchasing patterns and food outlet utilisation. Finally, this is the first longitudinal study from Australia that has investigated the simultaneous change over time in the local food environment and diet of residents. Therefore, replication in other longitudinal studies is needed.

## Figures and Tables

**Table 1:** Summary statistics of participant characteristics.

Participant Characteristics	T2 (*n* = 1453)		T3 (*n* = 1212)		T4 (*n* = 558)	
	Mean (SD)	*n* (%)	Mean (SD)	*n* (%)	Mean (SD)	*n* (%)
Age (years)	41.7 (11.8)		44.5 (11.8)		47.8 (11.9)	
Male		560 (38.5)		468 (38.6)		213 (38.2)
Education level:						
*Secondary or less/other*		564 (38.8)		442 (36.5)		196 (35.1)
*Trade/apprentice/certificate*		543 (37.4)		463 (38.2)		198 (35.5)
*Bachelor or higher*		346 (23.8)		307 (25.3)		164 (29.4)
Married/de facto ^1^		1237 (85.1)		1038 (85.6)		479 (85.8)
Hours of work per week:						
*Not working/no response*		313 (21.6)		258 (21.3)		126 (22.6)
*≤19*		178 (12.3)		146 (12.0)		89 (15.9)
*20–38*		374 (25.7)		332 (27.4)		151 (27.1)
*39–59*		530 (36.5)		421 (34.7)		175 (31.4)
*≥60*		58 (4.0)		55 (4.5)		17 (3.0)
Household income (AU$):						
*< 50,000/no response*		385 (26.5)		263 (21.7)		115 (20.6)
*50,000–69,999*		315 (21.7)		206 (17.0)		50 (9.0)
*70,000–89,999*		328 (22.6)		215 (17.7)		77 (13.8)
*≥90,000*		425 (29.2)		528 (43.6)		316 (56.6)
Children < 18 years at home ^1^		737 (50.7)		592 (48.8)		308 (55.2)
Access to a motor vehicle ^1^		1344 (92.5)		1139 (94.0)		523 (93.7)
Physical activity (hrs/week) ^2^	4.9 (5.1)		5.2 (5.5)		5.6 (5.6)	
BMI (kg/m^2^)	26.0 (4.8)		26.4 (4.9)		26.4 (4.5)	
Area-level SES (deciles)	8.0 (1.4)		8.3 (1.7)		8.3 (1.8)	

Abbreviations: BMI = body mass index; SES = socio-economic status. ^1^ Reference levels: married/de facto vs. separated/divorced/widowed/single/no response; yes always access to a motor vehicle vs. no/don’t drive/yes sometimes; yes children <18 years at home vs. no children < 18 years at home. ^2^ Total hours per week of walking/cycling for recreation/transport and moderate to vigorous leisure time physical activity.

**Table 2:** Summary statistics of dietary outcomes and predictor variables.

Study Variables	T2 (*n* = 1453)		T3 (*n* = 1212)		T4 (*n* = 558)	
	Mean (SD)	*n* (%)	Mean (SD)	*n* (%)	Mean (SD)	*n* (%)
***Dietary outcomes*** **^1^**						
Healthy diet	4.9 (1.6)		5.0 (1.5)		5.1 (1.6)	
Unhealthy diet	10.7 (2.6)		10.3 (2.8)		10.1 (2.9)	
Fruit/vegetable intake	4.1 (1.4)		4.1 (1.2)		4.1 (1.4)	
Diet quality	69.0 (7.0)		69.6 (6.8)		69.9 (6.9)	
***Individual behaviours*** **^2^**						
Frequency of eating meals bought from a canteen/takeaway food shop	2.8 (1.4)		2.6 (1.4)		2.4 (1.5)	
Frequency of eating meals bought from a restaurant/café	2.4 (1.2)		2.4 (1.2)		2.3 (1.2)	
***Perceptions of the local food environment*** **^3^**						
Presence of a café or restaurant within 15 min walk of home		355 (24.4)		405 (33.4)		264 (47.3)
Presence of a supermarket/greengrocer within 15 min walk of home		347 (23.7)		440 (36.3)		293 (52.5)
***Spatial exposure to the local food environment***						
**1.6 km road network buffer:**						
Count takeaway/fast food	1.0 (2.3)		1.4 (2.9)		2.4 (3.5)	
Count café restaurant	0.7 (3.1)		1.0 (1.2)		1.8 (5.1)	
Count convenience store	1.0 (1.2)		1.1 (1.6)		1.5 (1.9)	
Count supermarket/greengrocer	0.5 (1.2)		0.8 (1.6)		1.1 (1.8)	
MRFEI (%)	18.0 (27.5)		19.5 (26.0)		19.8 (21.0)	
**Road network distance to nearest (km):**						
Takeaway/fast food	2.3 (1.4)		2.2 (1.3)		1.7 (1.4)	
Café restaurant	2.5 (1.4)		2.3 (1.5)		2.0 (1.6)	
Convenience store	1.7 (0.9)		1.5 (0.7)		1.4 (0.9)	
Supermarket/greengrocer	2.8 (1.8)		2.4 (1.8)		2.0 (1.5)	

Abbreviations: MRFEI = modified retail food environment index (higher numbers mean a greater percentage of healthy food outlets). ^1^ Healthy diet scores ranged from 0–12 with higher scores reflecting a healthier diet; unhealthy diet scores ranged from 0–18 with higher scores reflecting an unhealthier diet; fruit/vegetable intake ranged from 0 = do not eat to 10 = 12 servings or more; and diet quality scores ranged from 0–100 with higher scores reflecting a better diet quality. ^2^ Individual behaviours ranged from 0 = never to 6 = 6–7 times per week. ^3^ Reference level = no.

**Table 3:** Single factor associations between changes in predictor variables and changes in dietary outcomes.

Predictor Variables	Healthy Diet ^1^		Unhealthy Diet ^1^		Fruit/vegetable Intake ^1^		Diet Quality ^1^	
	β	95% CI	β	95% CI	β	95% CI	β	95% CI
***Individual behaviours***								
Frequency of eating meals bought from a canteen/takeaway food shop	−0.09 ***	***−0.14, −0.05***	0.37 ***	***0.29, 0.45***	−0.10 ***	***−0.14, −0.05***	−0.67 ***	***−0.88, −0.46***
Frequency of eating meals bought from a restaurant/café	−0.05	−0.10, 0.01	0.29 ***	***0.20, 0.39***	−0.07 **	***−0.12, −0.02***	−0.52 ***	***−0.76, −0.28***
***Perceptions of the local food environment***								
Presence of a café or restaurant within 15 min walk of home ^2^	0.11	−0.01, 0.23	0.19	−0.02, 0.39	0.11 *	***0.001, 0.23***	0.21	−0.33, 0.75
Presence of a supermarket/greengrocer within 15 min walk of home ^2^	−0.01	−0.14, 0.11	0.20	−0.01, 0.41	0.02	−0.09, 0.13	−0.25	−0.80, 0.29
***Spatial exposure to the local food environment***								
**1.6 km road network buffer:**								
Count takeaway/fast food	0.003	−0.03, 0.03	0.01	−0.05, 0.06	−0.001	−0.03, 0.03	−0.07	−0.22, 0.07
Count café restaurant	0.01	−0.02, 0.05	−5.70 × 10^−5 3^	−0.05, 0.05	0.01	−0.02, 0.03	−0.01	−0.15, 0.13
Count convenience store	−0.03	−0.08, 0.02	−0.05	−0.14, 0.03	−0.03	−0.08, 0.02	−0.16	−0.38, 0.06
Count supermarket/greengrocer	0.03	−0.03, 0.09	−0.01	−0.10, 0.09	0.02	−0.03, 0.08	0.08	−0.18, 0.34
MRFEI (%)	0.003 *	***0.000, 0.01***	0.001	−0.004, 0.01	0.002	0.00, 0.01	0.01	−0.01, 0.02
**Road network distance to nearest (km):**								
Takeaway/fast food	0.01	−0.05, 0.07	0.02	−0.07, 0.11	−0.01	−0.06, 0.04	0.01	−0.23, 0.26
Café restaurant	0.04	−0.01, 0.10	−0.05	−0.14, 0.04	0.02	−0.02, 0.07	0.24 *	***0.01, 0.48***
Convenience store	0.06	−0.03, 0.14	0.08	−0.06, 0.22	0.06	−0.02, 0.14	0.09	−0.28, 0.46
Supermarket/greengrocer	0.002	−0.04, 0.05	−0.01	−0.08, 0.07	−0.004	−0.04, 0.04	0.02	−0.17, 0.21

Abbreviations: MRFEI = modified retail food environment index (higher numbers mean a greater percentage of healthy food outlets). ^1^ Adjusted for all time-varying participant characteristics and time. ^2^ Reference level = no. ^3^ Denotes −5.70 × 10^−5^. Significant results in ***bold italics***. * *p* ≤ 0.05, ** *p* ≤ 0.01, *** *p* ≤ 0.001.
